# Transport Evidence of Surface States in Magnetic Topological Insulator MnBi_2_Te_4_

**DOI:** 10.1021/acsnanoscienceau.5c00185

**Published:** 2026-03-27

**Authors:** Michael Wissmann, Romain Giraud, Börge Mehlhorn, Maxime Leroux, Mathieu Pierre, Michel Goiran, Walter Escoffier, Bernd Büchner, Anna Isaeva, Joseph Dufouleur, Louis Veyrat

**Affiliations:** † Leibniz Institute for Solid State and Materials Research, 28394 IFW Dresden, Helmholtzstrasse 20, 01069 Dresden, Germany; ‡ 129881 Université Grenoble Alpes, CNRS, CEA, Grenoble-INP, Spintec, 38000 Grenoble, France; § Würzburg-Dresden Cluster of Excellence ct.qmat, 01062 Dresden, Germany; ∥ CNRS, Laboratoire National des Champs Magnétiques Intenses, 131766 Université Grenoble-Alpes, Université Toulouse 3, INSA-Toulouse, EMFL, 31400 Toulouse, France; ⊥ Department of Physics, TU Dresden, 01062 Dresden, Germany; # Institute of Physics, University of Amsterdam, 1098 XH Amsterdam, The Netherlands; ∇ Faculty of Physics, Technical University of Dortmund, 44227 Dortmund, Germany; ○ Research Center “Future Energy Materials and Systems” (RC FEMS), 44227, Dortmund, Germany

**Keywords:** MnBi_2_Te_4_, Shubnikov-de Haas oscillations, Magnetic topological insulators, Topological surface states, High magnetic field, Magneto-transport, Nanostructures

## Abstract

Magnetic topological insulators can host chiral 1D edge channels at zero magnetic field, when a magnetic gap opens at the Dirac point in the band structure of 2D topological surface states, leading to the quantum anomalous Hall effect in ultrathin nanostructures. For thicker nanostructures, quantization is severely reduced by the coexistence of edge states with other quasi-particles, usually considered as bulk states. Yet, surface states also exist above the magnetic gap, but it remains difficult to identify electronic subbands by electrical measurements due to strong disorder. Here we unveil surface states in MnBi_2_Te_4_ nanostructures, using magneto-transport in very-high magnetic fields up to 55 T, giving evidence of Shubnikov-de-Haas oscillations above 40 T. A detailed analysis confirms the 2D nature of these quantum oscillations, thus establishing an alternative method to photoemission spectroscopy for the study of topological surface states in magnetic topological insulators using Landau level spectroscopy.

## Graphical abstract

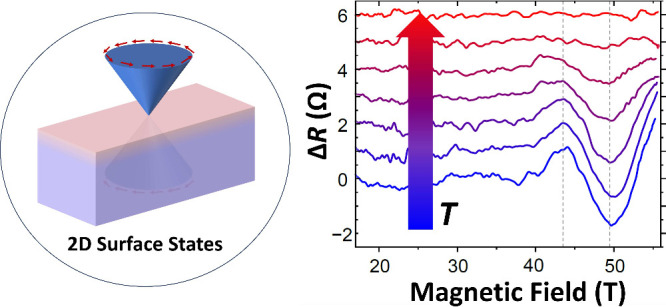

Topological insulators have metallic interface states at their boundaries, which can be either surface states for 3D systems, or edge states for 2D systems.^1–3^ These states are of great interest for applications in spintronics due to their helical spin textures with spin-momentum locking,^4,5^ and they are weakly scattered by disorder. In nonmagnetic 3D topological insulators, the anisotropic scattering of topological surface states (TSS) results in an increase of their backscattering length, as compared to other quasi-particles, which is associated with the enhanced transport mobility of TSS with respect to the mobility of bulk carriers^6^ and which is at the origin of quasi-ballistic transport in narrow quantum wires.^7–9^ In magnetic topological insulators, a small nontrivial gap opens in the 2D band structure of surface states due to the exchange field, and 1D ballistic edge states contribute to the quantum anomalous Hall effect.^10^ However, the quantization of the transverse resistance in ultrathin structures, important for quantum metrology,^11,12^ can be severely reduced by bulk carriers.^13^ Actually, in thicker nanostructures, a major limitation to having quantized transport properties could rather come from surface electronic states, which exist above the magnetic gap but within the bulk-band gap. However, due to strong disorder in magnetic TIs, it remains difficult to evidence the nature of electronic sub-bands by electrical measurements.

One convenient way to individually access the doping and energy levels of the surface and bulk electronic populations is to perform Landau level spectroscopy. The measurement of Shubnikov-de Haas oscillations (SdHO) of the resistance under external magnetic field can give access to the carrier density, the transport and the quantum mobilities, the effective mass, and the chemical potential of different charge carrier populations, whose contributions to the total conductance can be disentangled so. However, this technique requires magnetic fields such that μB > 1, μ being the charge carrier mobility, different for each band. While such quantum oscillations have been studied in Sb-doped MnBi_2_Te_4_,^14,15^ they were not observed in pure MnBi_2_Te_4_ in the many magneto-transport studies up to 15 T,^16–21^ except for the report of small resistance oscillations,^22^ which are not attributed to surface states, but to Weyl physics. This absence of SdHO in pure MnBi_2_Te_4_ is most probably due to a low mobility caused by the strong disorder in the bulk, attributed mainly to Mn/Bi antisites,^23–28^ thus requiring very high magnetic fields to observe SdHO.

Here, we report magneto-transport measurements in Hallbar nanostructures of exfoliated MnBi_2_Te_4_ up to very high magnetic fields (55 T) for the first time, revealing the clear signature of SdHO above 40 T. From the detailed study of their temperature and angular dependence, we identify the 2D nature of the electronic band responsible for these SdHO. By comparing the band-specific SdHO carrier density to the average carrier density extracted from the Hall effect, we determine the band bending of the bulk band at the top surface of the nanostructure. This study provides the first transport evidence of surface states in magnetic topological insulators.

We prepare our samples by exfoliating flakes from high-quality MnBi_2_Te4 single crystals grown by a controlled protocol developed by us in refs 23 and 28. The products of all growth experiments are routinely checked by powder X-ray diffraction (X’Pert Pro diffractometer (PANalytical), Bragg–Brentano geometry with a variable divergence slit, a curved Ge(111) monochromator, Cu–Kα_1_ radiation (λ = 154.056 pm) . Phase analysis of the growth batches is performed by Le Bail profile-fitting method in JANA2006, and shows that the samples are multiphase as expected for the incongruently melting MnBi_2_Te_4_, as explained in full detail in ref 28. The growth batches contain well-shaped platelet-like single crystals that are mechanically extracted and confirmed as MnBi_2_Te_4_ by energy-dispersive X-ray spectroscopy (see Figure S8 in the Supporting Information). Their average composition is Te 59.4(7) atom %, Bi 28.1(8) atom %, Mn 12.5(4) atom % in accordance with ref 28. These exact verified crystals are then used for further exfoliation.

The exfoliated flakes were then patterned using e-beam lithography to make ohmic Ti/Au contacts and a TiO_2_ hard mask to etch a Hall bar. We investigated two devices that yielded very similar results, as shown in the Supporting Information. An atomic force microscopy picture of one such device is presented in Figure 1a. The flake’s thickness is 91 nm, with a low surface roughness of about 1 nm. The temperature dependence of the longitudinal resistance shows a dirty-metal behavior (low residual resistance ratio), with a pronounced resistance peak at 25.5 K at the antiferromagnetic Néel transition temperature *T*_N_.

**Figure 1:**
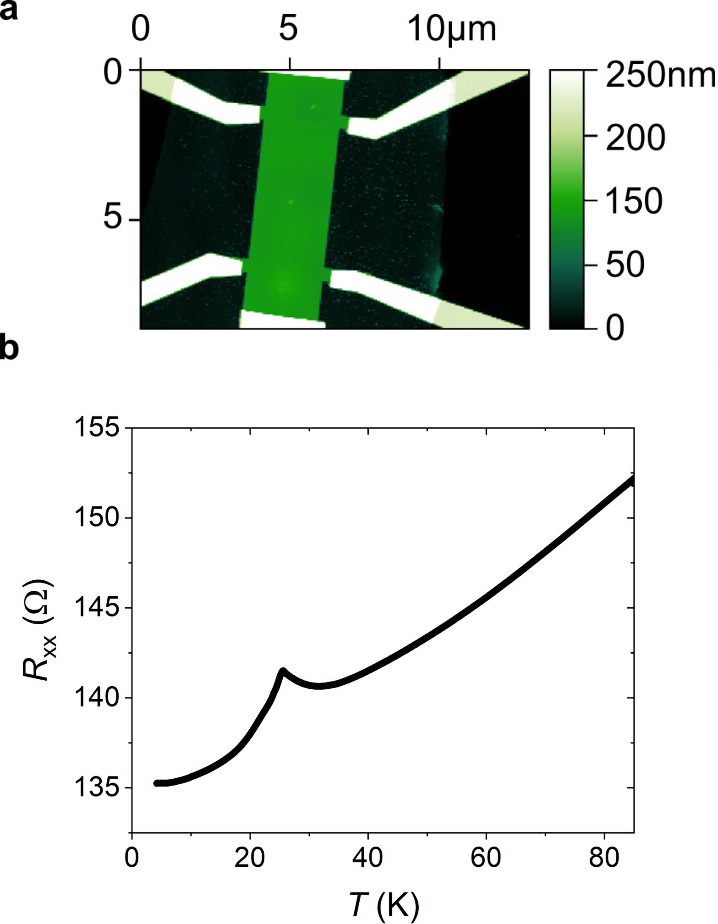
Device MBT2: Hall-bar geometry and metallic behavior. (a) Atomic force microscopy picture of an etched MnBi_2_Te_4_ nanostructure, with a thickness of 91 nm and roughness of about 1 nm. The original shape of the MnBi_2_Te_4_ exfoliated flake can still be faintly seen below the contacts. (b) Temperature dependence of the longitudinal resistance in zero magnetic field. The antiferromagnetic transition induces a resistance peak at 25.5 K.

Figure 2 presents the longitudinal and the transverse magneto-resistance under perpendicular magnetic fields up to 55 T. At low field, both samples show the expected signature of a collinear antiferromagnet with a uniaxial anisotropy: a sharp resistance peak at about 3.1 T corresponds to the spin-flop magnetic transition,^29^ which is also visible in the transverse resistance as a sharp jump of the anomalous Hall effect (blue arrows). Another feature is observed at 7 T, corresponding to the saturation field above which the magnetization is uniform (Supporting Information). This low-field magneto-transport, together with the observed Neel temperature, confirms that our nanostructures have all the usual magneto-transport properties expected of MnBi_2_Te_4_ samples. Above 10 T, the transverse resistance is linear up to 55 T, corresponding to an asymptote of the normal Hall effect, yielding a total bulk carrier density of 6.7 × 10^19^ cm^–3^.

**Figure 2:**
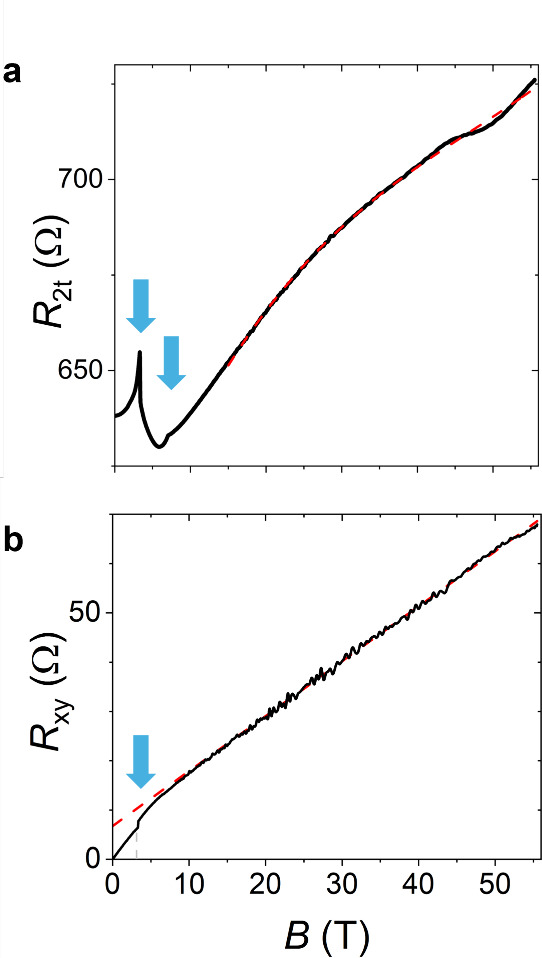
Device MBT1: magneto-resistance and Hall response in the out-of-plane configuration. The longitudinal magneto-resistance (a) and the transverse magneto-resistance (b) were measured at 4.2 K up to 55 T, and the data were symmetrized and antisymmetrized with respect to the magnetic field, respectively. Shubnikov-de Haas oscillations are visible above about 40 T. Purple dashed lines represent respectively a cubic fit of the magneto-resistance, used afterward to extract Shubnikov-de-Haas oscillations and a linear fit of the Hall effect asymptote at high field. Arrows indicate the spin-flop and saturation fields.

Above 40 T, oscillations appear in the longitudinal magneto-resistance. While only one and a half full oscillations are visible, those appear periodic in the inverse magnetic field, as can be seen in Figure 3a. Their amplitude increases with the magnetic field, and they are damped by increasing temperature while remaining at the same position in field. We therefore identify them as Shubnikov-de Haas oscillations. The appearance of SdHO at such high field reflects the moderate quantum mobility of the carriers, about 250 cm^2^/(V s). The periodicity in the inverse magnetic field of 0.003 T^–1^ corresponds to a magnetic frequency *f*_B_ = 167 T, a parameter directly related to the sub-band carrier density. We investigate the temperature dependence of the oscillations after removal of the monotonic background for each temperature, as shown in Figure 3b. The oscillations are slowly damped by increasing temperature, until they disappear at around 110 K. From this temperature dependence, we extract the effective mass m* of the corresponding charge carriers through a Lifshitz-Kosevich fit (see Figure 3c).^30^ The obtained value is m* = 0.16 ± 0.01 *m*_e_, which could be compatible with both bulk states (close to 0.15 *m*_e_: the effective mass of the second bulk conduction band CB2, but significantly different from the other two bulk bands, see below and^31^) or with topological surface states with linear energy dispersion (for which m* = *E*_F_/*v*_F_^2^, with *E*_F_ and *v*_F_ the Fermi energy and Fermi velocity).

**Figure 3:**
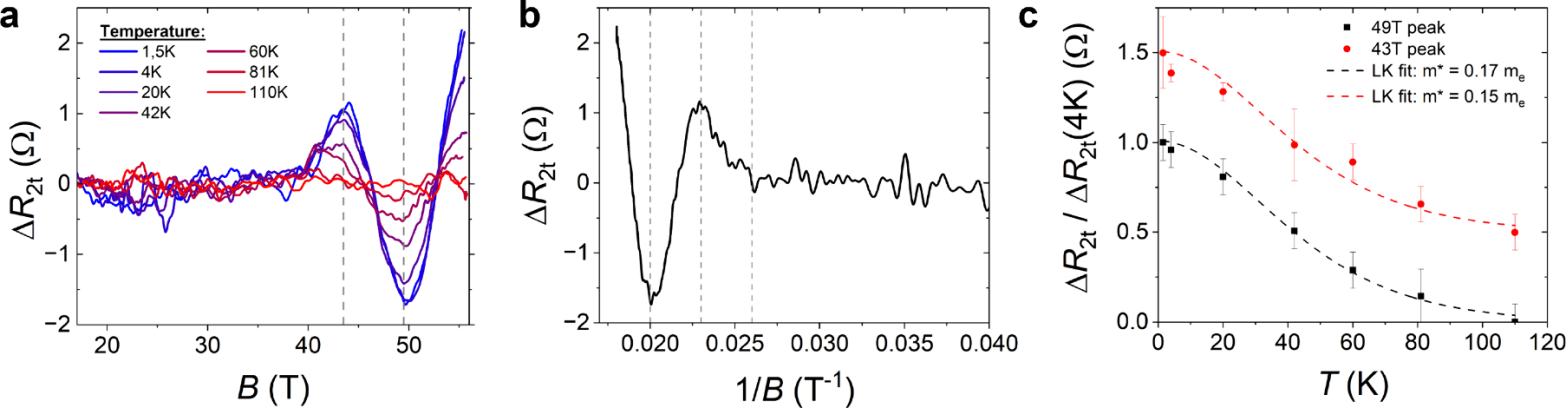
Evidence of Shubnikov-de Haas oscillations and Lifshitz-Kosevich analysis. (a) Residual Shubnikov-de Haas oscillations after removal of a cubic background, at different temperatures. The positions of the minimum and maximum of the oscillation are indicated by dashed lines. (b) Residual Shubnikov-de Haas oscillations after removal of a cubic background versus inverse magnetic field. The dashed lines show the positions of the SdHO extrema, separated by 0.003 T^–1^. (c) Temperature dependence of the Shubnikov-de Haas oscillation amplitude for the peaks at 43 and 49 T. The error bars correspond to rapid noise in the raw data. Lifshitz-Kosevich fits lead to effective masses m* of about 0.15 – 0.17 *m*_e_.

In order to determine the 3D or 2D character of the electronic population responsible for the observed SdHO, the magnetic field was tilted out of the sample plane by an angle of θ. From the angular dependence of the magneto-resistance, we observe that the SdHO evolves with the angle but is stable with respect to *B*_⊥_ = *B*cos (θ). Figure 4a shows the SdHO plotted against the perpendicular component of magnetic field *B*_⊥_. The position of the maximum and minimum of the visible SdHO remains unaffected, while the positions vary with regard to the total field. This is a clear sign that the SdHO originate from a 2D electronic state in the sample plane. This is also compatible with the carrier density extracted from the SdHO frequency: if interpreted as a 3D parabolic bulk band, a magnetic frequency *f*_*B*_ = 167 T corresponds to a bulk carrier density of *n*_3D_^SdHO^ = 1/2π^2^ (2*e*/*h* × *f*_B_)^3/2^ = 8 × 10^17^ cm^–3^, which is 2 orders of magnitude smaller than the total carrier density extracted from the Hall effect. Interpreting the SdHO as originating from a topological surface state (TSS) with linear energy dispersion and a Fermi velocity *v*_F_ = 5.5 × 10^5^ m s^–1^,^32,33^ we calculate a 2D carrier density of *n*_2D_^SdHO^ = e/*h* × *f*_B_ = 4.1 × 10^12^ cm^–2^ at the topological surface state, and a Fermi energy

$$E_{F}^{\mathrm{TSS}}=\hbar \cdot v_{F}\cdot \sqrt{4\pi \cdot n_{2D}^{\mathrm{SdHO}}}=255\;\mathrm{meV}$$

above the Dirac point in good agreement with ARPES.^34,35^

**Figure 4:**
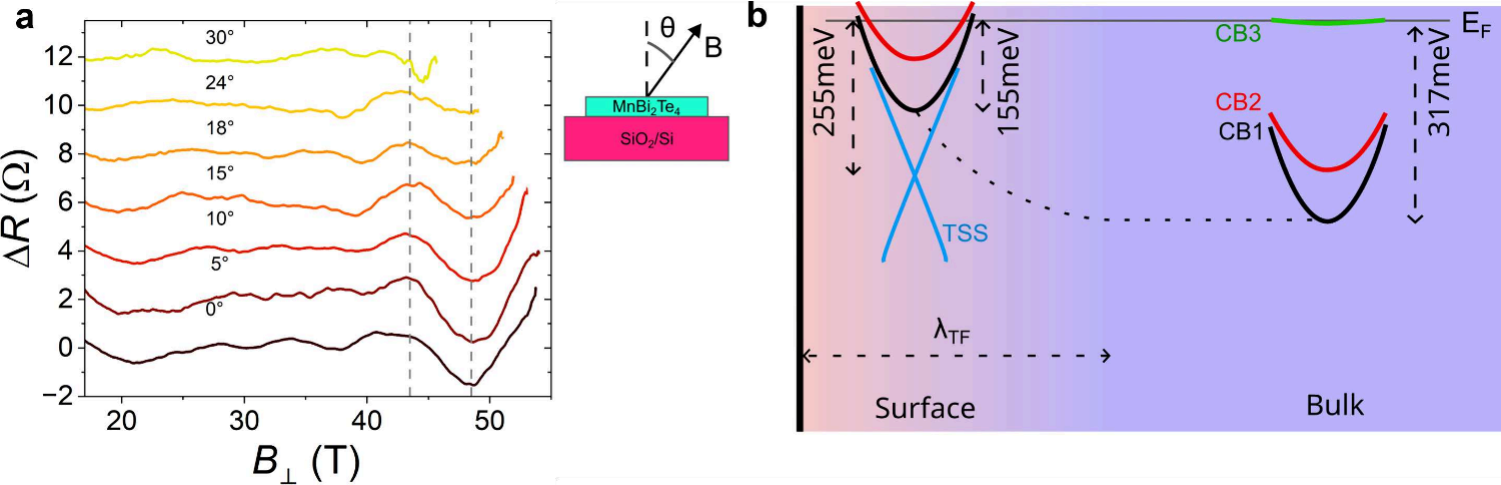
Angular dependence of Shubnikov-de Haas oscillations and band bending analysis. (a) Angular dependence of the Shubnikov-de-Haas oscillations after removal of a cubic background, between 0° (transverse field configuration) to 30° tilt toward in-plane configuration, shown against the transverse magnetic field component *B*_⊥_ = *B* cos­(θ). Curves were shifted for clarity. The schematic shows the tilting angle θ of the magnetic field. (b) Schematic of the energy levels and band bending in the MnBi_2_Te_4_ nanostructure, as extracted from Shubnikov-de Haas and Hall data (see main text), in a model with three conduction bands (CB1, CB2, and CB3, see main text) and one topological surface state (TSS).

In order to determine whether the surface state was localized on the top or the bottom surface of the nanostructure, we measured the dependence of the SdHO while applying a back-gate voltage (Supporting Information). As a large back-gate voltage of 110 V did not induce any change on the SdHO, we conclude that the surface state responsible for the observed SdHO is not located on the bottom surface, but rather on the top one. The reason for the absence of visibility of SdHO originating from a bottom surface state probably lies in a lower mobility, which could be due to interaction with charge traps in the SiO_2_ substrate.

Consequently, we draw a model of the band filling and bending in the nanostructure. This model, based on the density functional theory (DFT) calculations from ref 31, is summarized in Figure 4b and detailed below. DFT predicts three conduction bands in the bulk centered on the Γ point, CB1, CB2 and CB3, with respective effective masses 0.09 *m*_*e*_, 0.15 *m*_*e*_ and 3 *m*_*e*_, and bottom band energies at 80 and 310 meV below CB3.^31^ We used this simplified model of the bulk band structure to calculate the energy profile of the nanostructure at the bulk and surface. In this simple calculation, the bands are supposed to be perfectly parabolic and isotropic.

Since the carrier density of a 91 nm thick nanostructure is likely to be dominated by the bulk, we calculate the bulk carrier density as the total carrier density extracted from high-field Hall slope: *n*_3D_^Hall^ = 6.7 × 10^19^ cm^–3^. In the three-bulk-band model, this corresponds to a bulk Fermi level about 317 meV above the bottom of the conduction band CB1 (see Supporting Information). The bulk chemical potential is therefore pinned at the bottom of the very heavy third conduction band CB3. While using a more exact model based on integrated density of states from DFT would change the exact value of the calculated bulk chemical potential, we argue that given the very large carrier density measured, the main result of the model—the very large bulk chemical potential—would not change qualitatively.

We now model the chemical potential at the surface using the charge carrier density extracted from the SdH oscillations. As calculated above, the chemical potential of the surface lies 255 meV above the Dirac point. Using a separation of 100 meV between the Dirac point and the lowest conduction band CB1 from the literature (as determined for instance by ARPES, see refs 35–37 ), this imposes the position of the bulk band at the surface 155 meV below the Fermi energy. As can be seen in Figure 4, this results in an upward band bending of more than 150 meV between the bulk and surface. This upward band bending is a common situation in heavily doped 3DTI,^38^ where topological surface states are filled by charge transfer from heavily doped bulk bands, resulting in a lower doping of the bulk band close to the topological surface over the length-scale of the Thomas-Fermi screening length. It is, however, interesting to notice that, given the important band bending between TSS and bulk states, surface-sensitive techniques such as ARPES measurements would significantly underestimate the bulk chemical potential and in particular the filling of the third conduction band CB3. Given the large effective mass of CB3, the bulk chemical potential will efficiently be pinned at the bottom of CB3 for carrier densities larger than ∼ 5 × 10^19^ cm^–3^.

We can further check the self-consistency of our model by calculating the effective mass expected for such topological surface states with such a Fermi energy. Using m* = *E*_F_/*v*_F_^2^, we obtain m* = 0.148 *m*_e_, which is very close to the value extracted from the SdHO temperature dependence. This further confirms the 2D character of our SdHO. For TSS, the electronic mean free path is *l*_e_ = μm*v_F_/*e* = *μE*_F_/*ev*_F_. Considering the quantum mobility μ = 1/(40 T) = 250 cm^2^/(V s) from the SdHO onset, this gives a lower estimate of the electronic mean free path *l*_e_ ≃11 nm. This is lower than the value measured in nonmagnetic TIs like Bi_2_Se_3_ (21 nm,^6^), which is consistent with the higher doping level of MnBi_2_Te_4_ as well as with the stronger chemical disorder, as due to cation intermixing.

Summing up the results of analysis and discussion, the hypothesis of 2D surface state-origin SdH oscillations is supported by (a) the 1/cos angle dependence of the peak position in magnetic field and (b) the effective masses m* obtained from the model being consistent with the ones extracted from the Lifshitz-Kosevich fit of the measured SdH oscillations. This band bending model as obtained from the 3-band DFT model under the 2D hypothesis is further consistent with band bending reported on other topological insulators,^38^ and the surface chemical potential estimated from *f*_B_ is also consistent with ARPES results.^34,35^

In conclusion, we have studied magneto-transport up to very high magnetic fields in Hallbars of exfoliated MnBi_2_Te_4_. Above 40 T we evidence clear Shubnikov-de Haas oscillations due to the moderate mobility of the charge carriers in MnBi_2_Te_4_. The angular dependence of the Shubnikov-de Haas oscillations in tilted field reveals their 2D origin. From Shubnikov-de Haas and Hall effect analysis we construct a model of band bending in our nanostructure, yielding properties of both bulk and surface states. Our study presents the first transport evidence of surface states in MnBi_2_Te_4_, which should play an important role in determining the transport properties in thin nanostructures. In particular, understanding and controlling the properties of the topological surface states are important to optimize the quantum anomalous Hall effect in magnetic topological insulators.

## Supplementary Material



## Notes

Additional magneto-transport data on both MnBi_2_Te_4_ devices, including gate-, angle- and field dependencies (PDF)
