# Supplementary material for: Transport Evidence of Surface States in Magnetic Topological Insulator MnBi2Te4
Source: ACS Nanosci Au. 2026 Mar 27;6(3):357–62. doi: 10.1021/acsnanoscienceau.5c00185 (PMC13281201; doi:10.1021/acsnanoscienceau.5c00185)
Supplement: Supplementary file 1 [file ng5c00185_si_001.pdf]

Transport evidence of surface states in magnetic topological insulator $MnBi_2Te_4$

Michael Wissmann,[1,2] Romain Giraud,[1,2] Börge Mehlhorn,[1,3] Maxime Leroux,[4] Mathieu Pierre,[4] Michel Goiran,[4] Walter Escoffier,[4] Bernd Büchner,[1,3,5] Anna Isaeva,[6,7] Joseph Dufouleur,[1,3] and Louis Veyrat[4]

[1]*Leibniz Institute for Solid State and Materials Research, IFW Dresden, Helmholtzstrasse 20, 01069 Dresden, Germany*
[2]*Université Grenoble Alpes, CNRS, CEA, Grenoble-INP, Spintec, 38000 Grenoble, France*
[3]*Würzburg-Dresden Cluster of Excellence ct.qmat, 01062 Dresden, Germany*
[4]*CNRS, Laboratoire National des Champs Magnétiques Intenses, Université Grenoble-Alpes, Université Toulouse 3, INSA-Toulouse, EMFL, 31400 Toulouse, France*
[5]*Department of Physics, TU Dresden, 01062 Dresden, Germany*
[6]*Institute of Physics, University of Amsterdam, 1098 XH Amsterdam, The Netherlands*
[7]*Faculty of Physics, Technical University of Dortmund, 44227 Dortmund, Germany and Research Center "Future Energy Materials and Systems" (RC FEMS), 44227, Dortmund, Germany*

SUPPLEMENTARY INFORMATION

1. ADDITIONAL MAGNETOTRANSPORT DATA ON MBT1

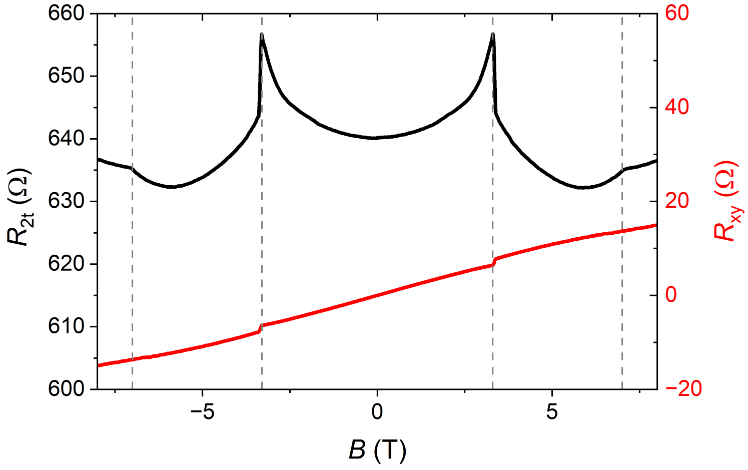

FIG. S1. **Low field magnetotransport in sample MBT1.** Zoom-in on the low-field (up to 8T) two-terminal R_{2t} magnetoresistance (black) and antisymmetrized transverse resistance (red), measured for a magnetic field applied perpendicular to the sample plane, showing the transport signatures of magnetic transitions in $MnBi_2Te_4$. The position of the spin-flop transition (±3.3T) and saturation field (±7T) is highlighted by dashed lines.

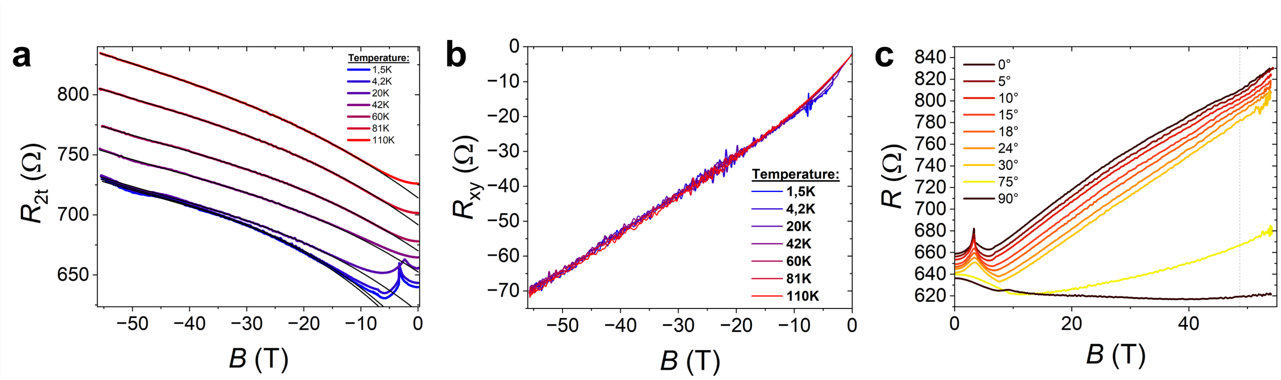

FIG. S2. **Raw Data, sample MBT1 (main text). a:** Two-terminal magnetoresistance up to 55T, measured at several temperatures, measured for a magnetic field applied perpendicular to the sample plane. The black lines are the cubic fit used to extract ΔR_{2t} presented in the main text.**b:** Corresponding Hall resistance $R_{\rm xy}$. **c:** Two-terminal magnetoresistance measured at 4.2K for different tilting angle θ. The curves are vertically shifted for clarity. The position of the Shubnikov-de-Haas minimum at 0° is highlighted by a dotted line. No SdHO is visible with in-plane field (90°).

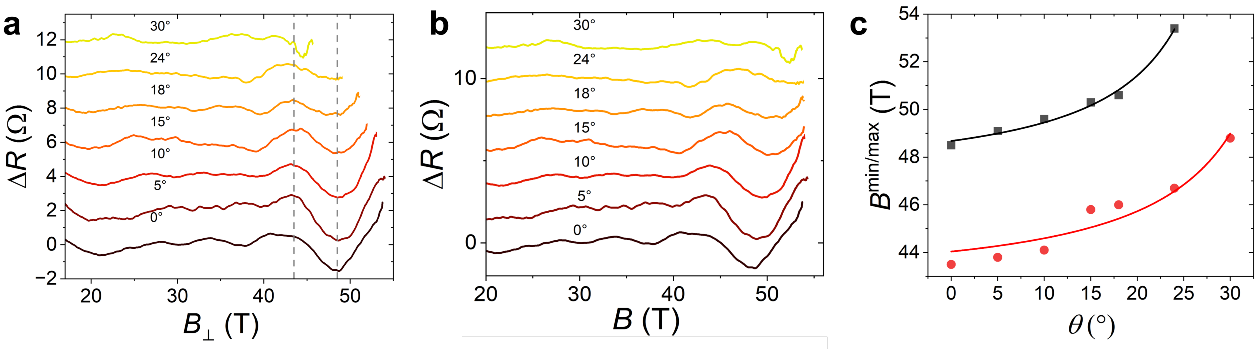

FIG. S3. **Angle-dependence of the SdH oscillations. a:** SdH oscillations for various angles in dependence of the transverse component of the applied magnetic field. Minima and maxima appear for all angles at the same fields, indicated by the grey dashed lines. this figure corresponds to Fig. 4 a) in the main text. **b:** The same SdH oscillations as in a), in dependence of the total applied magnetic field. minima and maxima appear at higher total field values for increasing angles. **c:** Angular dependence of the magnetic field positions at which minimum and maximum of the SdH oscillation appear (squares and circles, respectively). The data is appropriately fitted by a $1/\cos(\theta)$- function (lines).

2. SHUBNIKOV-DE-HAAS OSCILLATIONS REPRODUCED IN MBT2

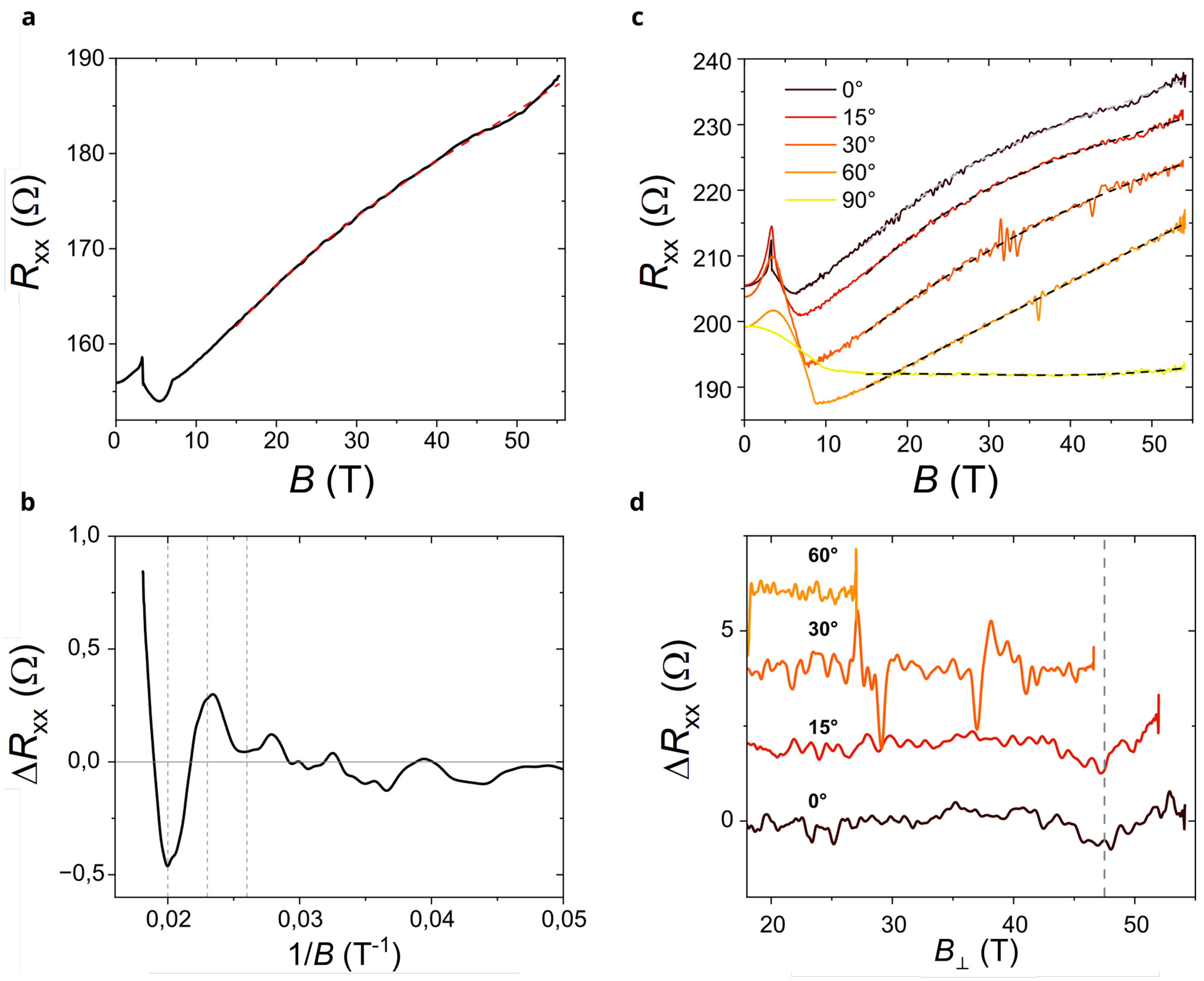

FIG. S4. **Shubnikov-de-Haas oscillations in second sample MBT2. a:** Four-terminal longitudinal R_{xx} magnetoresistance up to 55T with perpendicular magnetic field. The red dashed line is the cubic fit used to extract ΔR_{xx} presented in panel **b**. **b:** Residual magnetoresistance ΔR_{xx} from panel **a** after removing a cubic background. SdHO very similar to that of sample MBT1 are observed. **c:** Magnetoresistance measured at 4.2K and up to 55T for different tilting angle θ. The position of the Shubnikov-de-Haas minimum at 0° is highlighted by a dotted line. No SdHO is visible with in-plane field (90°). The dashed lines are the cubic fit used to extract ΔR_{xx} presented in panel **d**. **d:** Angular dependence of Shubnikov-de-Haas oscillations after removal of a cubic background, between 0° (perpendicular field configuration) to 60° tilt toward in-plane configuration, shown against the perpendicular magnetic field component. The curves are vertically shifted for clarity. The position of the minimum highlighted by a dashed line is stable at 0° and 15°, and disappears above 30°, where the maximum perpendicular magnetic field component is below the SdHO minimum position. This supports the 2D nature of this SdHO, similar as for MBT1 presented in the main text.

3. MODEL OF BAND STRUCTURE

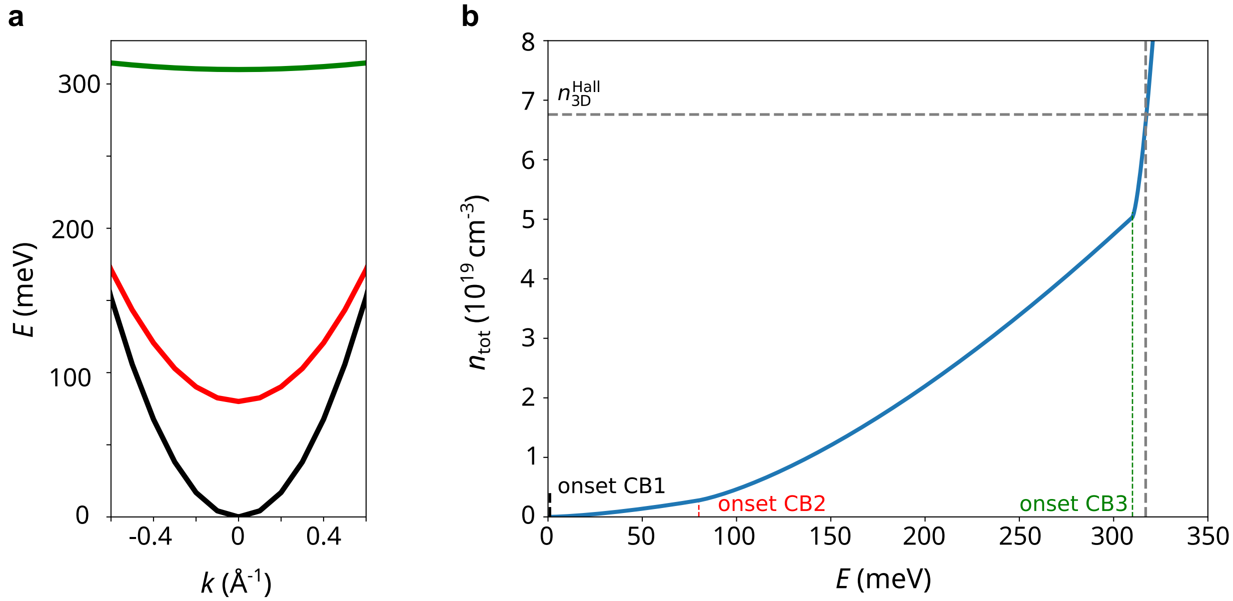

FIG. S5. **Model of the 3-bulk-band structure and carrier density calculated. a:** Band structure modeled from the DFT calculations of [31]. The onset of the three conduction bands CB1, CB2 and CB3 are 0, 80meV, and 310meV, with effective masses 0.09 m_e, 0.15 m_e and 3 m_e, respectively (see main text). **b:** Carrier density calculated from the 3-bulk-band model under the assumption of perfectly isotropic, parabolic bands, with knowledge of the effective masses from **a**. From the measured Hall carrier density, one extracts a bulk chemical potential of 317meV.

4. TRANSPORT UNDER APPLIED GATE VOLTAGE

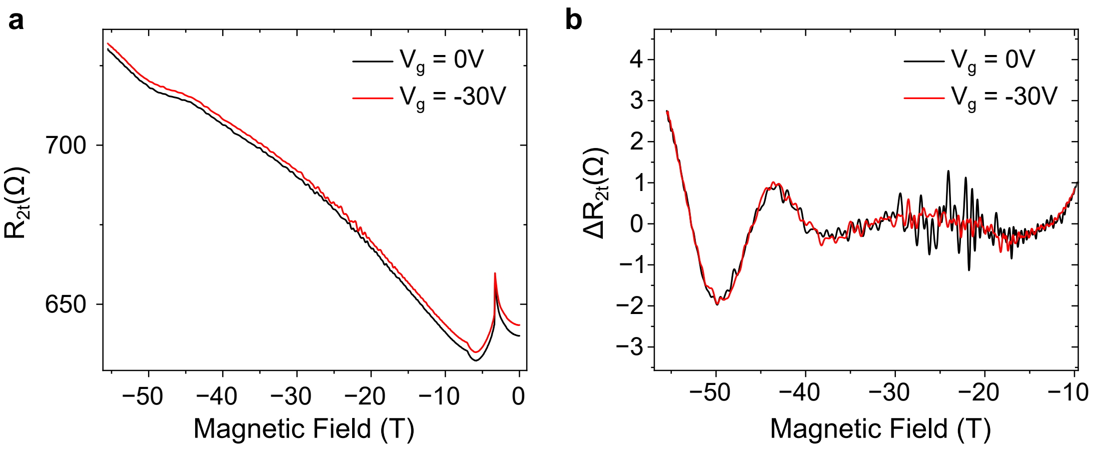

FIG. S6. **Absence of gate voltage dependence of the Shubnikov-de-Haas oscillations. a:** Magnetoresistance of the two-terminal resistance of sample MBT1 for two values of the back-gate voltage V_g applied on the silicium wafer. **b:** Shubnikov-de-Haas oscillations after removal of a cubic background from the data of panel **a**. No variation is visible with the back-gate voltage.

To identify the surface state (top or bottom) responsible for the SdH oscillations, we applied a gate voltage to the Si substrate. Given the thickness of our SiO_2 gate dielectric (d =285nm) with dielectric constant $\epsilon \approx 3.5$, the surface capacitance of our device is $C_{\rm surf} = \epsilon_0 \cdot \epsilon / d = 10.8{\rm nF/cm}^2$. Using a gate voltage $V_{\rm g}$ = 30V, one induces a gate a surface charge of $Q = n_{\rm 2D} \cdot e = C_{\rm surf} \cdot V_{\rm g}$, so $n_{\rm surf} = C_{\rm surf} \cdot V_{\rm g}/e = 2 \cdot 10^{12}{\rm cm}^{-2}$. Given that the 2D charge carrier density extracted from SdH is $n_{\rm 2D}^{SdH} = 4.1 \cdot 10^{12}{\rm cm}^{-2}$, if it would originate from a the lower surface state, a backgate voltage of 30V would change the density (and hence the SdH frequency and period) by a factor 2, which would definitely be detectable in our measurement. As can be observed in Fig. S6, we observe no noticeable change in the SdH oscillations with $V_{\rm g}$ = -30V. As a result, we conclude that the SdH oscillations do not originate from the bottom surface state (at the interface with SiO_2), and consequently come from the top one. This analysis is also compatible with previous studies on topological insulators, see for instance refs. [6,38] of the main text.

5. AFM IMAGES OF THE TWO DEVICES

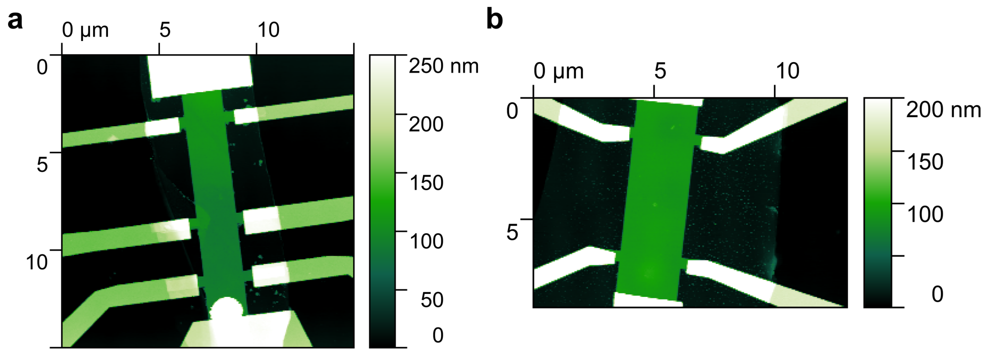

FIG. S7. **Atomic Force Microscopy images** of **a:** MBT1 (85nm/105nm thick) and **b:** MBT2 (91nm thick).

6. SINGLE CRYSTAL CHARACTERIZATION

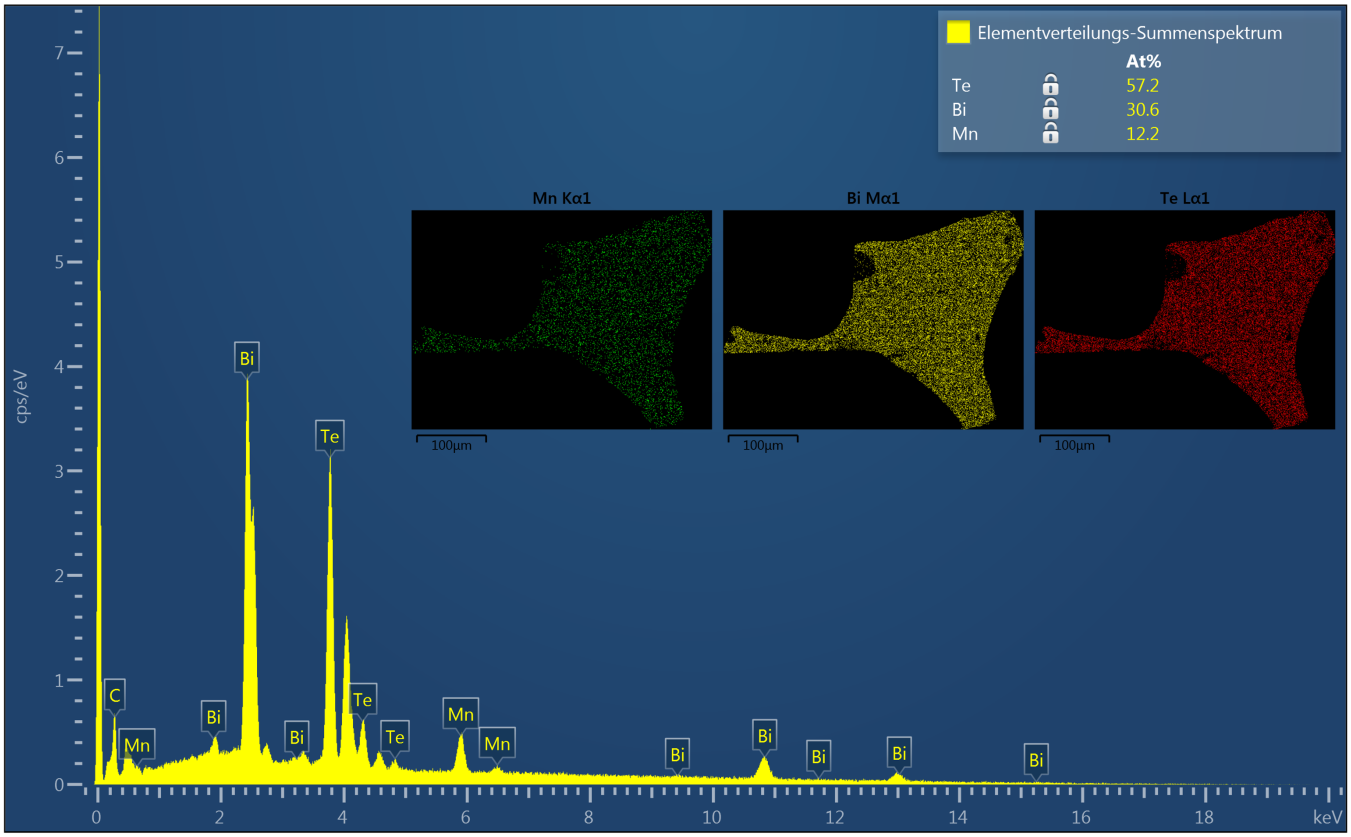

FIG. S8. EDS (energy dispersive x-ray spectroscopy) mapping on a selected single crystal, collected on a SU8020 (Hitachi) microscope equipped with a X-Max^N (Oxford) Silicon Drift Detector (SDD) at $U_a = 2 - 5\,\mathrm{kV}$ at 20 kV acceleration voltage. The derived composition corresponds to the chemical formula $Mn_{0.9(1)}Bi_{2.1(1)}Te_4$ in full accordance with the results of Ref. [28]
